# Comparative analysis of LytS/LytTR-type histidine kinase/response regulator systems in γ-proteobacteria

**DOI:** 10.1371/journal.pone.0182993

**Published:** 2017-08-10

**Authors:** Stefan Behr, Sophie Brameyer, Michael Witting, Philipp Schmitt-Kopplin, Kirsten Jung

**Affiliations:** 1 Munich Center for Integrated Protein Science (CIPSM) at the Department of Microbiology, Ludwig-Maximilians-Universität München, Martinsried, Germany; 2 Helmholtz Zentrum München, Deutsches Forschungszentrum für Gesundheit und Umwelt (GmbH), Research Unit Analytical BioGeoChemistry, Neuherberg, Germany; 3 TU München, Wissenschaftszentrum Weihenstephan für Ernährung, Landnutzung und Umwelt, Lehrstuhl für Analytische Lebensmittelchemie, Freising, Germany; Universidad de Costa Rica, COSTA RICA

## Abstract

Bacterial histidine kinase/response regulator systems operate at the interface between environmental cues and physiological states. *Escherichia coli* contains two LytS/LytTR-type histidine kinase/response regulator systems, BtsS/BtsR (formerly YehU/YehT) and YpdA/YpdB, which have been identified as pyruvate-responsive two-component systems. Since they exhibit remarkable similarity, we analyzed their phylogenetic distribution within the γ-proteobacteria, and experimentally characterized them in a set of representative species. We found that BtsS/BtsR is the predominant LytS/LytTR-type two-component system among γ-proteobacteria, whereas YpdA/YpdB primarily appears in a supplementary role. Based on our observations in *E*. *coli*, we used the highly conserved DNA-binding motifs to test the *in vivo* functionality of both systems in various genera, including *Salmonella*, *Enterobacter*, *Citrobacter*, *Xenorhabdus*, *Yersinia*, *Aeromonas* and *Vibrio*. The results suggest that, in all cases tested, BtsS/BtsR and YpdA/YpdB respond to different levels of pyruvate in the environment.

## Introduction

Prototypical bacterial two-component systems (TCSs) consist of a membrane-anchored sensory histidine kinase (HK) and a cognate response regulator (RR) with DNA-binding activity, and convert external environmental cues into an intracellularly mediated response [1]. This permits bacteria to rapidly adapt to changing environmental conditions and precisely control a variety of processes such as metabolism, stress responses or pathogenicity [2]. Here we focus on the LytS/LytTR-type HKs/RRs in γ-proteobacteria. HKs of the LytS family (family HPK8 in the classification of [3]) contain a common 5TM-5TMR_LYT domain (pfam07694), and are anchored in the membrane by at least five membrane-spanning domains. Moreover, they can show unusual N- and F-boxes in their kinase domains [3]. LytTR-type RRs belong to a family of transcriptional activators that contain a 10-stranded β-fold DNA-binding domain, instead of the more common helix-turn-helix domain [4–6]. Members of LytS/LytTR-type HK/RR systems can be found in many bacterial phyla and often control virulence and virulence-associated factors in human and plant pathogens [4]. *Escherichia coli* harbors two LytS/LytTR-type HK/RR systems: BtsS/BtsR (formerly named YehU/YehT) and YpdA/YpdB [7,8].

**Fig 1:**
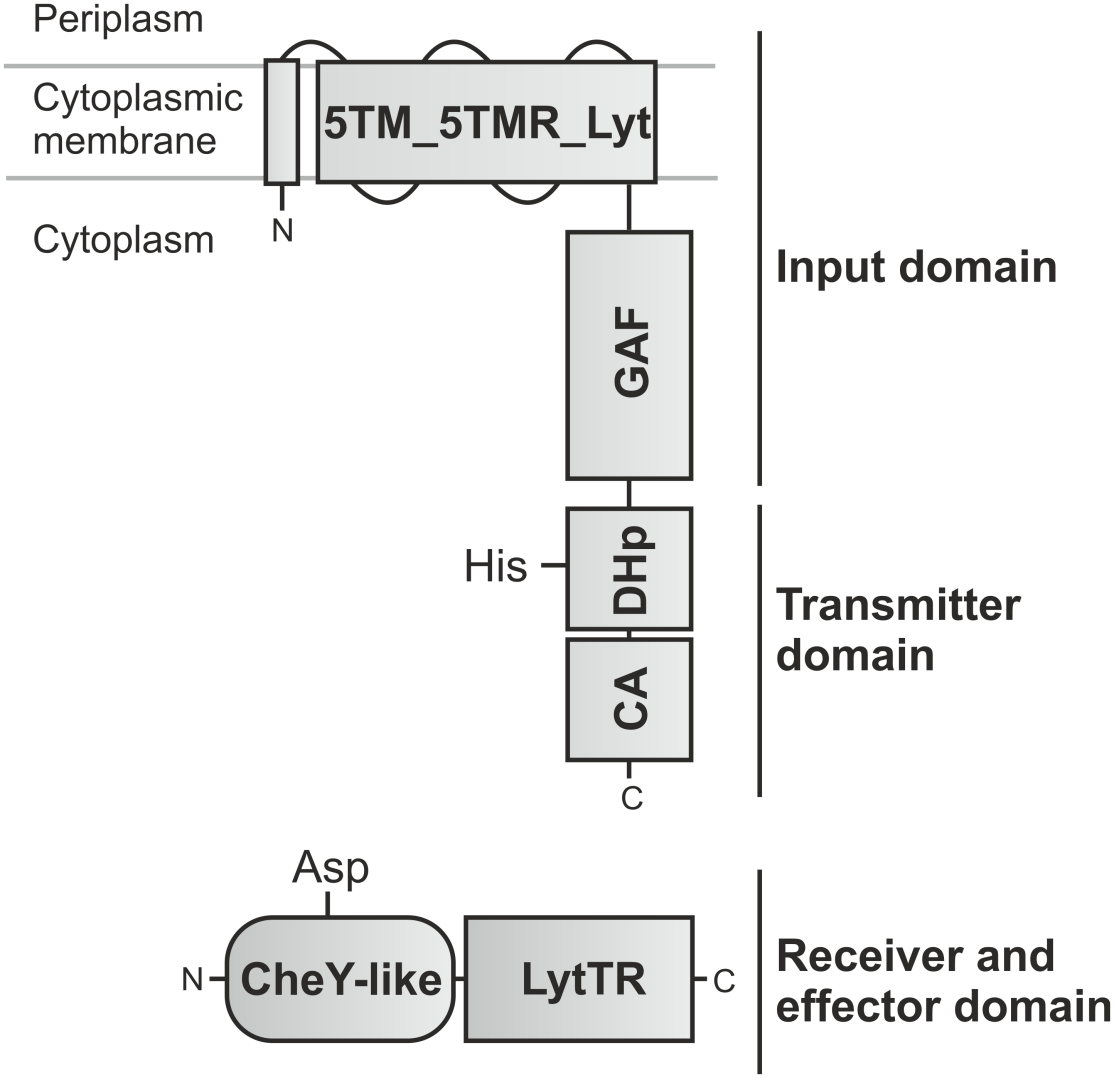
Domain organization of LytS/LytTR histidine kinase/response regulator. HKs of the LytS family contain a 5TM-5TMR_LYT domain (pfam07694) and a GAF domain, which together form the input domain. The cytosolic DHp [harboring a conserved histidine (His)] and CA modules in the HKs serve as the transmitter domain. The RR belongs to the LytTR-type family of transcriptional activators and comprises a CheY-like receiver domain [harboring a conserved aspartate (Asp)], and a LytTR-type effector domain made up of a 10-stranded β-fold DNA-binding domain.

Under nutrient-limiting conditions, in the presence of external pyruvate BtsS/BtsR activates the expression of *yjiY*, which encodes a CstA-like transport protein [9]. BtsS recognizes specifically and with high affinity pyruvate, which triggers signal transduction, and results in YjiY production [10]. The second LytS/LytTR-type HK/RR found in *E*. *coli*, YpdA/YpdB, regulates the expression of *yhjX*, coding for a putative antiporter of the major facilitator superfamily [7]. YhjX is produced whenever cells are cultivated in media containing a minimum concentration of 600 μM pyruvate [7]. Interestingly, the two systems are over 30% identical at the amino acid sequence level and share the same domain organization. The HKs each possess a C-terminal, membrane-integrated 5TM-5TMR_Lyt domain, linked to a cytosolic element containing GAF, DHp and CA domains, while the RRs consist of a CheY-like receiver domain connected to a LytTR-type DNA-binding (effector) domain [7,10] (Fig 1). Furthermore, the two systems are functionally interconnected. While YpdA/YpdB activity promotes BtsS/BtsR-mediated *yjiY* expression, activation of BtsS/BtsR down-regulates the YpdA/YpdB-mediated *yhjX* response under certain conditions [8]. In this study, we present a comprehensive and detailed overview of the phylogenetic distribution of BtsS/BtsR and YpdA/YpdB within the γ-proteobacteria. In addition, we assessed pyruvate-dependent signaling by both systems in a representative set of species, and developed an integrative model to explain their patterns of distribution and redundancy.

## Materials and methods

### Bioinformatics studies

Non-redundant LytS-type HKs were identified by Protein BLAST search, using the full-length amino acid sequence of *E*. *coli* BtsS (double-checked and complemented with YpdA) to query the RefSeq protein database [11], with an Expect (E) value < 1x10^-58^ and an amino acid length tolerance of 10% as default parameters. To elucidate the relationship between the LytS homologs, all non-redundant sequences were aligned and a phylogenetic tree was generated using CLC Main Workbench 7.6 (CLC Bio Qiagen, Hilden, Germany). To create the multiple sequence alignment, a progressive alignment algorithm from CLC Main Workbench 7 was used, which implements pairwise alignments beginning with the most similar pair and progressing to the most distantly related [12]. The tree was created using CLC’s high-accuracy, distance-based method with the neighbor-joining algorithm, with 100 bootstrap replicates and the Jukes-Cantor distance correction as default parameters [13]. The branch lengths therefore represent the degree of evolutionary divergence between any two nodes in the tree.

Putative targets were identified by local alignment search with the binding sequences targeted by *E*. *coli* YjiY and YhjX respectively, and additional analyses of the cognate DNA promoter sequences.

### Strains and plasmids

All bacterial strains used in this study are wild-type isolates available from the DSMZ (Braunschweig, Germany) and are listed in Table 1. Plasmids pBBR *yjiY*-*lux* and pBBR *yhjX*-*lux*, respectively, are promoter-based luciferase reporter constructs based on the corresponding target genes of *E*. *coli* MG1655 and were described in earlier studies [7,10].

**Table 1:** List of all tested strains. Gene names as indicated are taken from the NCBI database and represent LytS-type histidine kinase/response regulator systems and their putative target genes. The abbreviations (Ab.) listed are used in the further figures.

Strains	Ab.	BtsS/BtsR-type TCS; putative target gene *yjiY*	YpdA/YpdB-type TCS, putative target gene *yhjX*
*Escherichia coli* K-12 MG1655	*Ec*	*yehU-yehT* (renamed *btsS-btsR*); *yjiY*	*ypdA-ypdB*, *yhjX*
*Salmonella enterica* ser. Typhimurium SL1344	*Se*	*yehU-yehT*; SL1344_4463 (*cstA1*)^1^	Absent
*Enterobacter aerogenes* KCTC2190	*Ea*	EAE_23775–23780; EAE10255	EAE_00255–00260; EAE_06010
*Citrobacter freundii* ATCC 8090	*Cf*	D186_16097–16102; D186_06040	D186_12842–12837; D186_07871
*Xenorhabdus szentirmaii* DSM 16338	*Xs*	XSR_250031–250032; XSR_190061	Absent
*Serratia marcescens* ATCC 13880	*Sm*	GSMA_01246–01247; GSMA_00424	GSMA_02299–02298; GSMA_02296
*Yersinia enterocolitica* 8081	*Ye*	YE4014, YE0849; YE1981	YE1228-1227; YE1226^1^
*Aeromonas hydrophila* ATCC 7966	*Ah*	1) AHA_2618–2619; AHA_2620^1^ 2) *lytS*-AHA_3291; AHA_3292^1^	AHA_0066–0067; AHA_0068^1^
*Vibrio harveyi* ATCC 33867 [14]	*Vh*	M892_07980–07985; M892_07975^1^	Absent

^1^Target gene in close proximity to TCS genes.

### *In vivo* expression studies

*In vivo* promoter activities of *yhjX* and *yjiY* were quantified by means of luciferase-based reporter gene assays. For this purpose, all tested strains were transformed with pBBR *yjiY*-*lux* or pBBR *yhjX*-*lux*, respectively. Strains were cultivated overnight in lysogeny broth (LB; 10 g/l NaCl, 10 g/l tryptone, 5 g/l yeast extract) supplemented with 50 μg/ml gentamycin sulfate at 37°C (for *E*. *coli* and *S*. *enterica*) or 30°C (for the remaining strains). The optical density at 600 nm (OD_600_) as well as the luminescence levels [relative light units (RLU) expressed in counts per second per ml per OD_600_] were determined in a microplate reader (Tecan Infinite F500). Cultures, inoculated at a starting OD_600_ of 0.05, were grown in LB or M9 minimal medium with 0.4% (wt/vol) pyruvate as C-source and the appropriate antibiotic, at the indicated temperature under agitation, while OD_600_ and luminescence were monitored continuously over time.

### Determination of extracellular pyruvate

Each of the tested strains was inoculated to an OD_600_ = 0.05 in LB medium and aerobically grown at either 30°C or 37°C, as described above. At selected time points (0, 60, 90, 120, 180, 240, 300 and 360 min), the supernatants were harvested by centrifugation, and subsequently analyzed by hydrophilic interaction liquid chromatography (HILIC). Acetonitrile (ACN), ammonium acetate and ammonium hydroxide (all of LC-MS grade) were obtained from Sigma-Aldrich (Sigma-Aldrich GmbH). Water was purified using a Merck Millipore Integral water purification system to a resistance of 18 MΩ and TOC < 5 ppb. Sodium pyruvate was obtained from Sigma-Aldrich, dissolved in water at a concentration of 100 mM and further diluted with ACN. Supernatants were mixed with ACN (1:1) and transferred to autosampler vials.

Pyruvate excreted into the culture medium was quantified by a modified version of a previously published method [15]. Separation was achieved using a Waters XBridge Amide column (3.5 μm, 100 mm x 4.6 mm ID) and an ACN/ water gradient using a Waters Acquity UPLC system coupled to a Bruker maXis UHR-ToF-MS (Bruker Daltonics). Eluent A consisted of 5% (vol/vol) ACN, 95% (vol/vol) water, 20 mM ammonium hydroxide, 20 mM ammonium acetate, pH 9.0 and eluent B was pure ACN.

Metabolites were eluted as follows. After 3 min of 85% eluent B, a linear decrease to 2% B was applied over 9 min, followed by an isocratic hold for 3 min, return to initial conditions for 1 min and a 7-min re-equilibration time. Sample (5 μl) was injected via partial loop injection.

For quantification, a calibration curve was constructed using various concentrations of pyruvate (0, 0.5, 1.0, 2.5, 5, 10, 25, 50, 100 and 250 μM). Quantification was performed with Quant Analysis 4.4 (Bruker Daltonics).

## Results

### Identification and classification of γ-proteobacterial LytS-type histidine kinases

**Fig 2:**
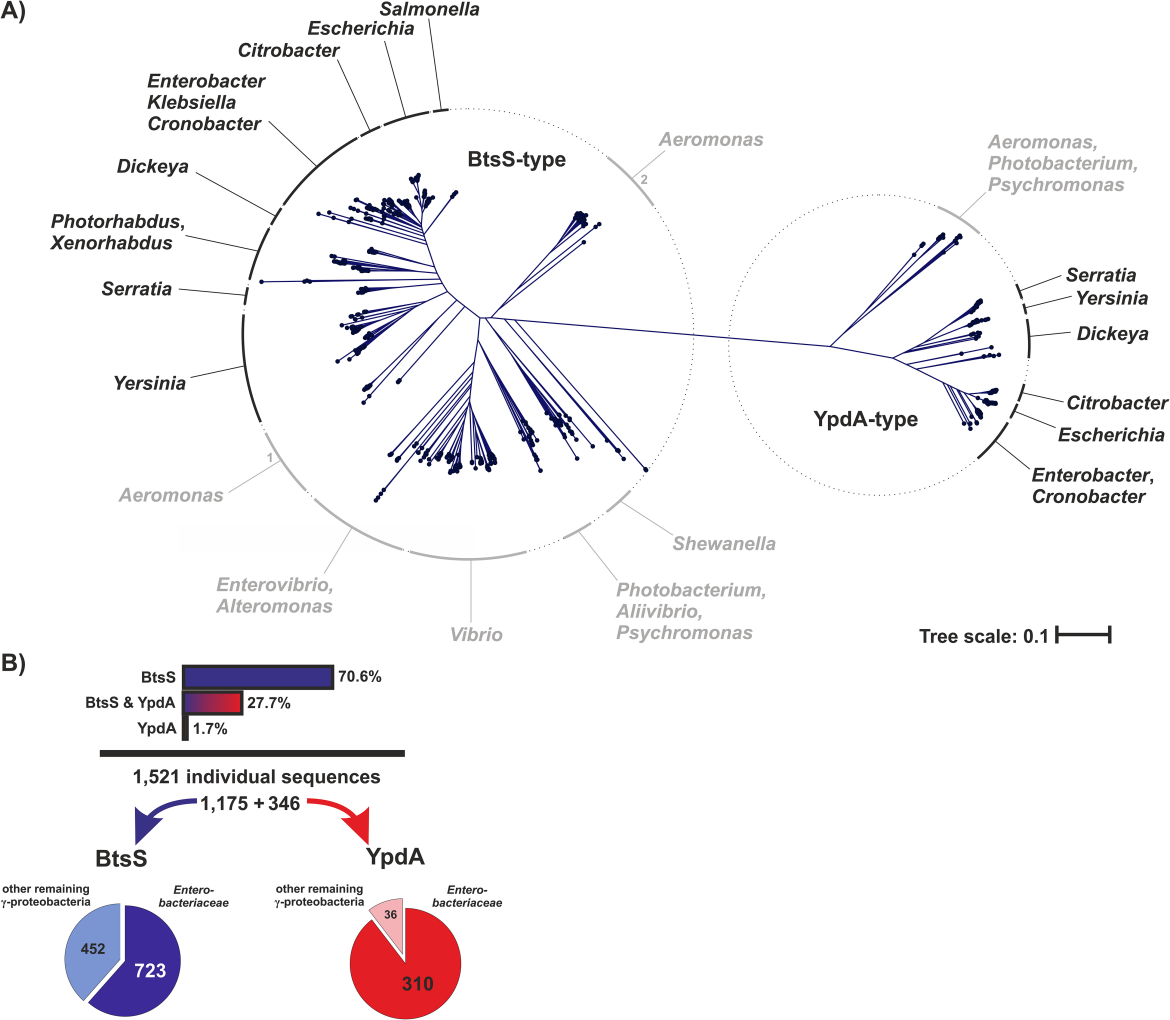
Phylogenetic tree of LytS-type histidine kinases in γ-proteobacteria. (**A**) Based on a local alignment search using the NCBI RefSeq protein database, 1,521 individual LytS-type sequences were identified, and amino acid identity was calculated using the progressive alignment algorithm of the CLC Main Workbench 7.6 software. The phylogenetic tree was created using CLC’s high-accuracy neighbor-joining algorithm with 100 bootstrap replicates. Reassigned genera are given in black for the predominant members of the *Enterobacteriaceae* and in grey in the case of other γ-proteobacterial families. All genes encoding BtsS- or YpdA-type proteins in the set of species included in the tree are listed in S1 Table. The lengths of the branches of the tree represent the relative amount of evolutionary divergence between any two sequences in the tree. (**B**) Summary of the relative abundance of BtsS and YpdA sequences detected, and their distribution among *Enterobacteriaceae* and other γ-proteobacteria based on the phylogenetic tree.

LytS-type HKs have the characteristic 5TM-5TMR_Lyt (LytS-YhcK)-type membrane-spanning input domain [16]. In order to identify LytS-type HKs from γ-proteobacteria, we performed a full-length amino acid sequence-based local alignment search using Protein BLAST [11]. Based on the alignment of 1,521 individual sequences (derived from a set of 18,523 redundant hits) we generated a phylogenetic tree and reassigned and grouped the corresponding bacterial genera (Fig 2A). The two known and well characterized LytS-type HKs in *E*. *coli*, BtsS and YpdA, were then used as branch markers, which also provided a reliable demarcation line. The majority (77.3%) of the sequences recovered were assigned to the (left) BtsS-type branch of the tree. The remaining 22.7% were YpdA-type HKs and are represented on the right-hand branch. While some genera, like *Escherichia*, *Citrobacter* or *Serratia*, harbor both BtsS and YpdA, others, including *Salmonella*, *Photorhabdus* or *Vibrio* have only one homolog, which is almost invariably assigned to the BtsS-type branch (Fig 2A). Interestingly, we also found species, especially in the genus *Aeromonas*, which possess two quite distinct BtsS-type HKs, which are also represented in different branches (Fig 2A). In the next step, we reassigned the data obtained to the original strains so as to discriminate between the different HKs within a given genome and check for co-occurrence. The majority (70.6%) of the strains were found to harbor only BtsS homologs. In 27.7% of all organisms, both BtsS and YpdA types could be identified, while a very small fraction of only 1.7% possessed the YpdA-type only (Fig 2B). Overall, two-thirds of the identified LytS-type HKs were found among the *Enterobacteriaceae*. Of the BtsS-type HKs, 38.5% belonged to organisms outside the enterobacteriaceal family, while the corresponding figure for the YpdA type is 10.4%.

Although the primary sequences of BtsS and YpdA share over 30% identity in *E*. *coli*, all identified LytS-type HKs could be unambiguously assigned to one or other of these categories (Fig 2A). BtsS-type HK is the most abundant LytS-type HK within the γ-proteobacteria (98.3%) and in the majority of species it is the only form present. In contrast, YpdA is less widely represented and almost always occurs together with BtsS (Fig 2A).

### Regulation of *yjiY* and *yhjX* genes in a set of representative species

**Fig 3:**
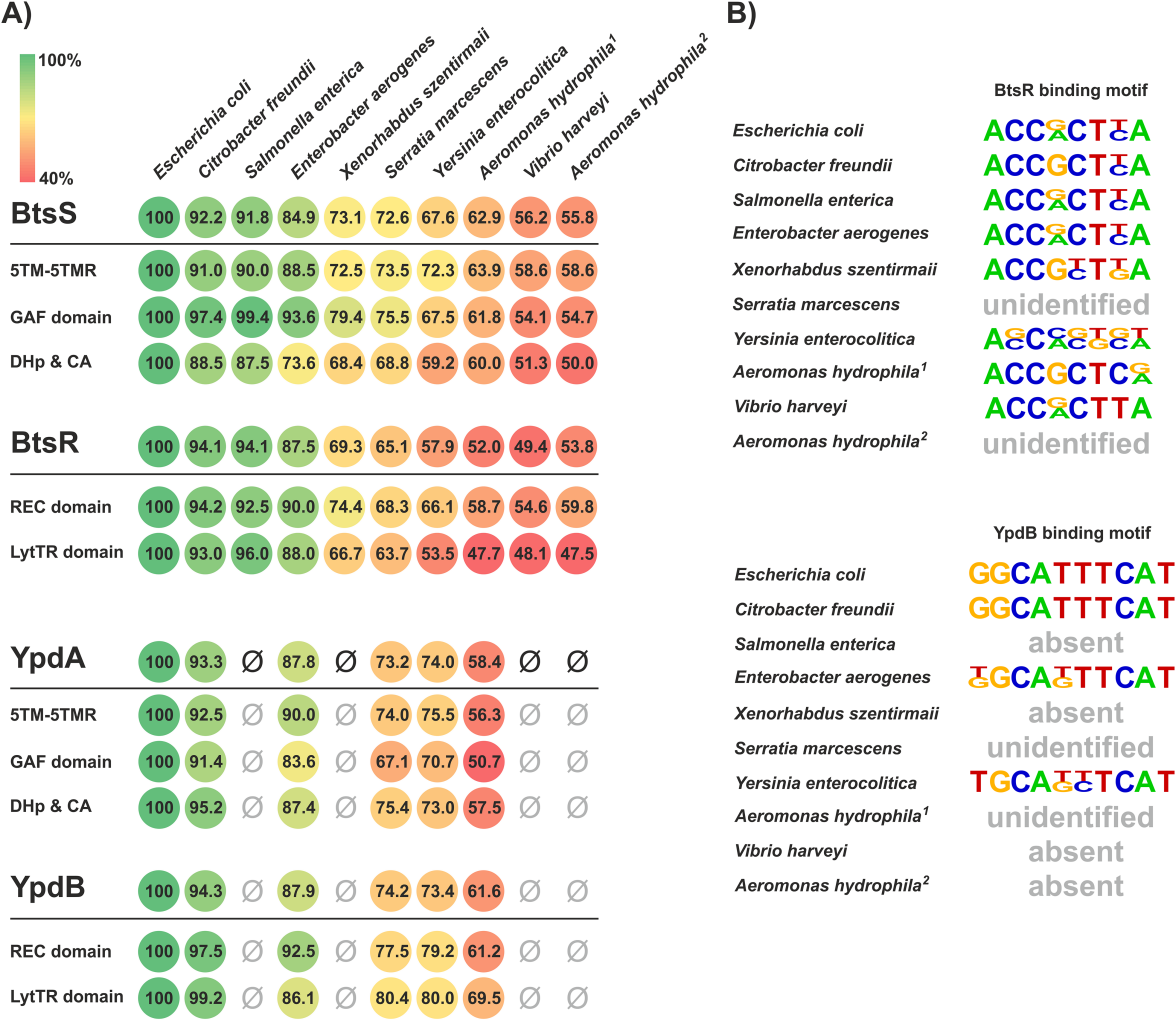
Comparison of BtsS/BtsR and YpdA/YpdB pairs in a subset of representative γ-proteobacterial species. (**A**) Full-length TCS proteins as well as their individual domains were aligned and analyzed for percentage of sequence identity with respect to the *E*. *coli* homologs (100%, green). The absence of YpdA/YpdB is marked with ‘Ø’. (**B**) In addition, promoter sequences of the identified target genes (Table 1) were scanned and RR-binding motifs were deduced. No YpdB target promoter motifs were detected in species in which the YpdA/YpdB-type TCS was either absent or could not be unambiguously identified and are marked with ‘absent’. Species, in which the known DNA-binding motif of BtsR or YpdB was undetectable, are marked with ‘unidentified’.

The classification of the identified LytS-type HKs into BtsS- and YpdA-type HKs was based solely on sequence comparisons. In the next step, we analyzed the reliability of this prediction by testing the functionality of these systems in eight representative species (Table 1). The primary sequences of BtsS and YpdA, respectively, as well as their corresponding RRs, BtsR and YpdB, were aligned and analyzed for the levels of identity within individual domains (Fig 2B). Levels of amino acid identity relative to the *E*. *coli* homologs generally declined in the more phylogenetically remote genera (Fig 3A). Nevertheless, the percentage of amino acid identity in all protein domains was found to exceed 50%, and was even higher in the GAF domain of BtsS [e.g. *Citrobacter freundii* (97.4%), *Salmonella enterica* (99.4%) and *Enterobacter aerogenes* (93.6%) in comparison to *E*. *coli*] (Fig 3A). Strikingly, no YpdA-type homologs were found in *Salmonella*, *Xanthomonas*, *Vibrio* or *Aeromonas*.

Furthermore, corresponding target genes could be identified for each system (Table 1), so each promoter region was scanned for the DNA-binding motifs known to be recognized in *E*. *coli* by BtsR or YpdB (Fig 3). Strikingly, in the majority of the species analyzed, BtsR- and YpdB-binding motifs were detectable within their putative *yjiY* and *yhjX* targets, and exhibited marked similarity to the known *E*. *coli* template sequences (Fig 3B). However, we were unable to identify binding motifs in the promoters of the corresponding target genes in *S*. *marcescens* or *A*. *hydrophila*. Although *A*. *hydrophila* harbors two BtsS/BtsR-type TCSs and one YpdA/YdpB-type TCS, their target genes and motifs seem to differ from those in *E*. *coli* (Fig 3B).

In light of the high degree of conservation, the selected species were transformed with the luciferase-based promoter reporter plasmids pBBR *yjiY*-*lux* and pBBR *yhjX*-*lux*, harboring the *yjiY* and *yhjX* promoter regions of *E*. *coli*, respectively [7,10]. Subsequently, we assayed for promoter activation upon exposure of these strains to inducing conditions. BtsS/BtsR-mediated activation of the *yjiY* promoter is coupled to nutrient limitation and the concurrent availability of a very low concentration of pyruvate in the medium (<5 μM), a condition that can be brought about by cultivating bacteria in amino acid-rich (LB) medium until the end of exponential growth. Under this condition *yjiY* expression is transiently induced [9]. In contrast, expression of *yhjX*, which is controlled by the YpdA/YpdB TCS, is activated in cells which are exposed to external pyruvate concentrations of at least 600 μM, and expression then lasts as long as pyruvate is available [7].

**Fig 4:**
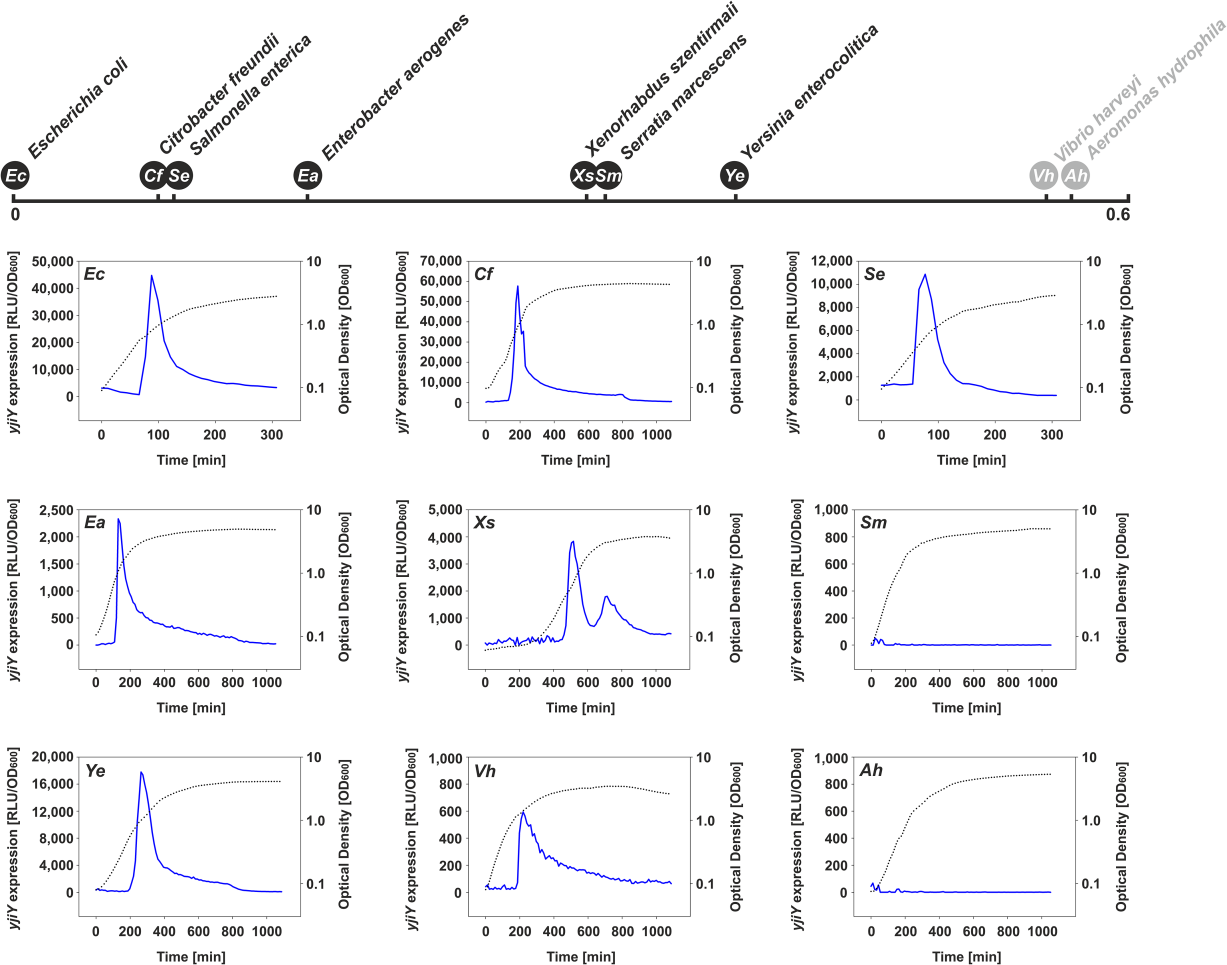
Activation of the *yjiY* promoter in various γ-proteobacterial species growing in LB medium. Previous studies reported transient BtsS/BtsR-mediated *yjiY* promoter activation in *E*. *coli* grown in LB medium shortly before cells enter stationary phase [9]. Each species was transformed with an *E*. *coli yjiY-lux* reporter plasmid, and luminescence levels (blue) as well as growth (black dotted line) were measured over time. *Escherichia coli* (*Ec*), *Citrobacter freundii* (*Cf*), *Salmonella enterica* (*Se*), *Enterobacter aerogenes* (*Ea*), *Xenorhabdus szentirmaii* (*Xs*), *Serratia marcescens* (*Sm*), *Yersinia enterocolitica* (*Ye*), *Vibrio harveyi* (*Vh*) and *Aeromonas hydrophila* (*Ah*). Degrees of divergence of the BtsS-type proteins of the selected species (based on phylogenetic tree in Fig 2A) to the *E*. *coli* BtsS are indicated above the graphs (in black—members of the *Enterobacteriaceae*, in grey—members of other γ-proteobacterial families).

**Fig 5:**
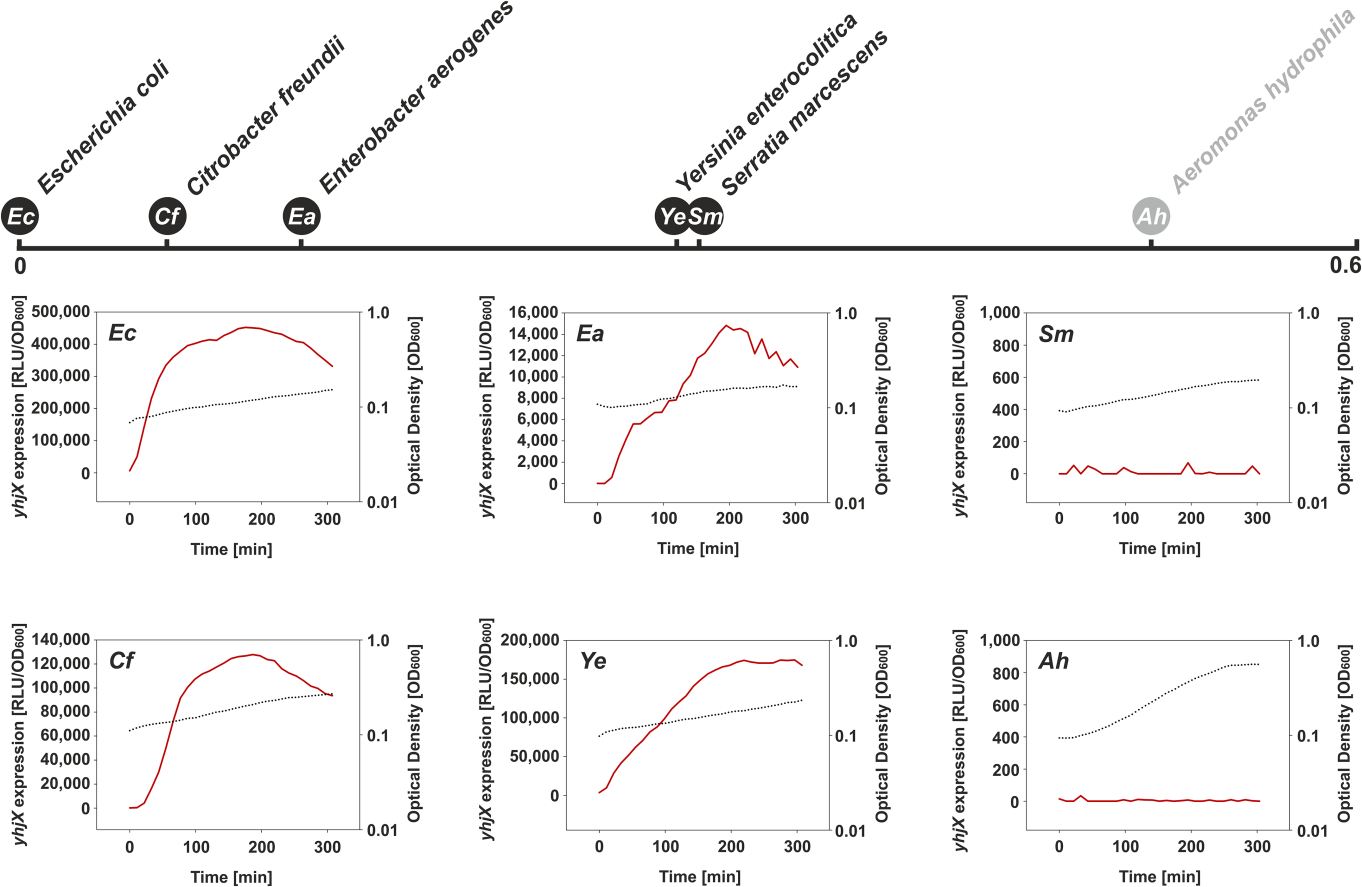
Activation of the *yhjX* promoter in selected γ-proteobacterial species grown in minimal medium containing 0.4% (wt/vol) pyruvate as C-source. Previous studies have reported constitutive YpdA/YpdB-mediated activation of the *yhjX* promoter in *E*. *coli* cultivated in minimal medium supplemented with pyruvate [7]. Each species was transformed with an *E*. *coli yhjX-lux* reporter plasmid, and luminescence levels (red) as well as growth (black dotted line) were measured over time. For further details, see the legend to Fig 4.

In all selected strains cultured under the above-mentioned conditions, the expression pattern(s) of the *yjiY* (Fig 4) and/or *yhjX* promoter(s) (Fig 5) basically mimicked those of *E*. *coli*. Only *S*. *marcescens* (*Enterobacteriaceae*) and *A*. *hydrophila* (*Aeromonadaceae*) failed to induce *yjiY* or *yhjX* promoter activity (Figs 4 and 5), most probably due to major rearrangements within the DNA-binding motifs of the putative target-gene promoter(s), as described before (Fig 3). Based on these results, it can be concluded that the functionality of both systems is maintained in most species of the *Enterobacteriaceae*. The lack of response in *Serratia* might be due to the use of the *E*. *coli*-based reporter plasmid, in which the BtsR- or YpdB-DNA binding motifs clearly differ from their native equivalents (see Fig 3B).

**Fig 6:**
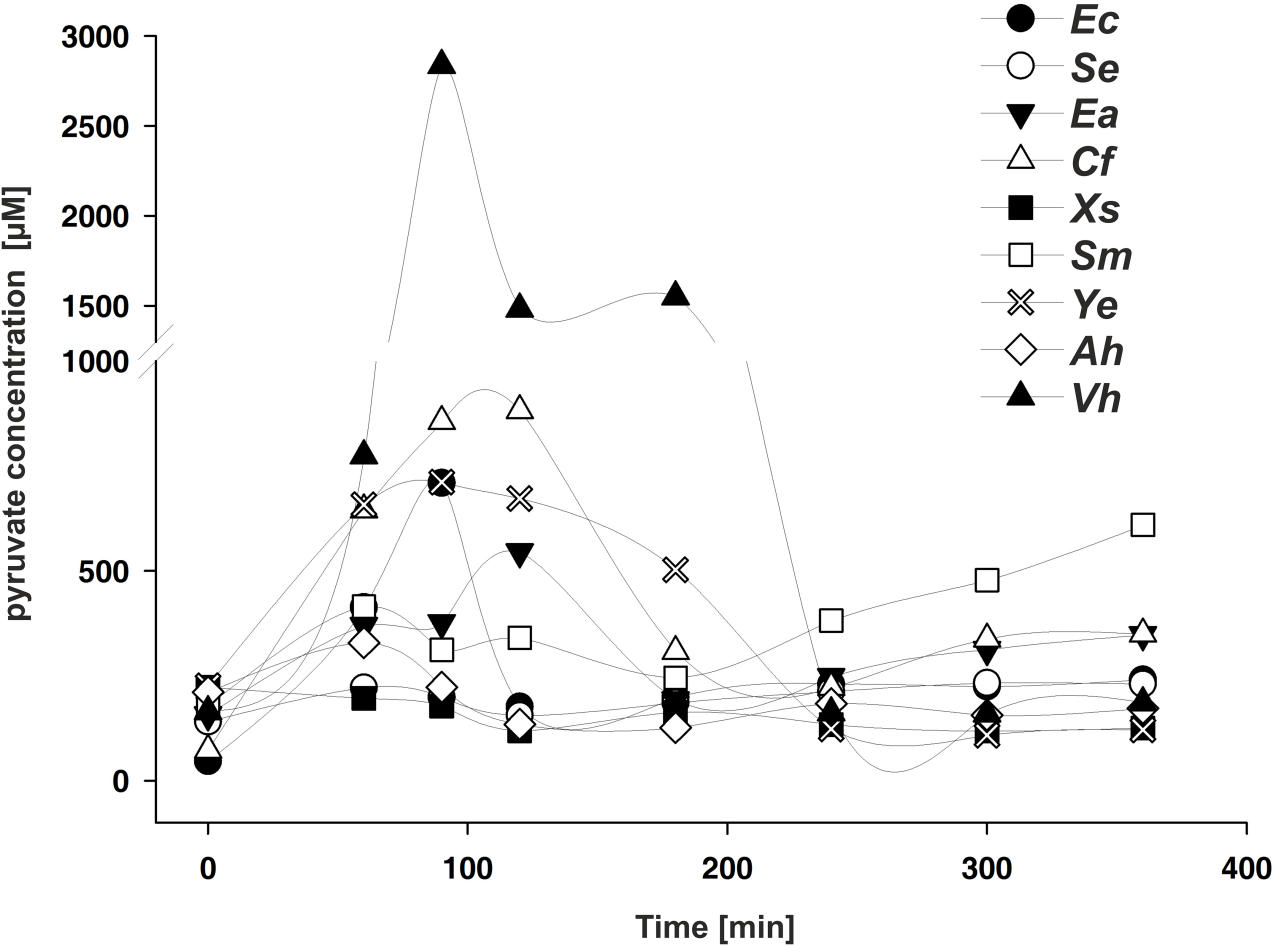
Extracellular concentrations of pyruvate during growth of the indicated γ-proteobacterial species in LB medium are plotted against time after inoculation. At the times indicated, cells were harvested, and pyruvate levels in the cell-free supernatant were quantified by hydrophilic interaction liquid chromatography. All experiments were performed in triplicate, and the error bars indicate the standard deviations of the means. Abbreviations as in Fig 4.

It was previously demonstrated that *E*. *coli* excretes pyruvate during growth in LB medium, and the onset of accumulation coincides with the activation of BtsS/BtsR and YpdA/YpdB [7]. Therefore, in the next experiment we tested whether other species that harbor these types of TCSs also excrete pyruvate during growth in an amino acid-rich medium, such as LB medium. For all tested bacteria we were able to detect external pyruvate at a minimal concentration of about 220 μM (Table 2). In addition, for C. *freundii*, *E*. *aerogenes*, and *Y*. *enterocolitica* we observed a transient accumulation of external pyruvate, as in *E*. *coli* (Fig 6). *S*. *marcescenes* released threefold less pyruvate than *E*. *coli* at the onset of the post-exponential growth phase, and a further increase was detectable in stationary phase (Fig 6). We did not find significant accumulation of external pyruvate under the tested conditions for *A*. *hydrophila*, *X*. *szentirmaii* and *S*. *enterica* (Fig 6). Notably *V*. *harveyi* exhibited an exceptionally strong release of pyruvate into the medium, although this species lacks the YpdA/YpdB-type TCS. These data suggest that the release of pyruvate during growth in LB medium, probably as part of the overflow metabolism, varies among **γ**-proteobacteria, but that under all conditions sufficient pyruvate is available to be sensed by the high affinity BtsS/BtsR-type system.

**Table 2:** Maximal accumulation of external pyruvate in the supernatant during growth in LB medium. Quantitative measurement of external pyruvate concentrations of all selected strains grown in LB medium (largest values out of one independent experiment). Plots of extracellular pyruvate concentrations over time are displayed in Fig 6.

Strains	Max. pyruvate concentration
*Escherichia coli* K-12 MG1655	709 μM
*Salmonella enterica* ser. Typhimurium SL1344	221 μM
*Enterobacter aerogenes* KCTC2190	544 μM
*Citrobacter freundii* ATCC 8090	881 μM
*Xenorhabdus szentirmaii*	222 μM
*Serratia marcescens* ATCC 13880	609 μM
*Yersinia enterocolitica* 8081	709 μM
*Aeromonas hydrophila* ATCC 7966	328 μM
*Vibrio harveyi* BA1115	2837 μM

## Discussion

**Fig 7:**
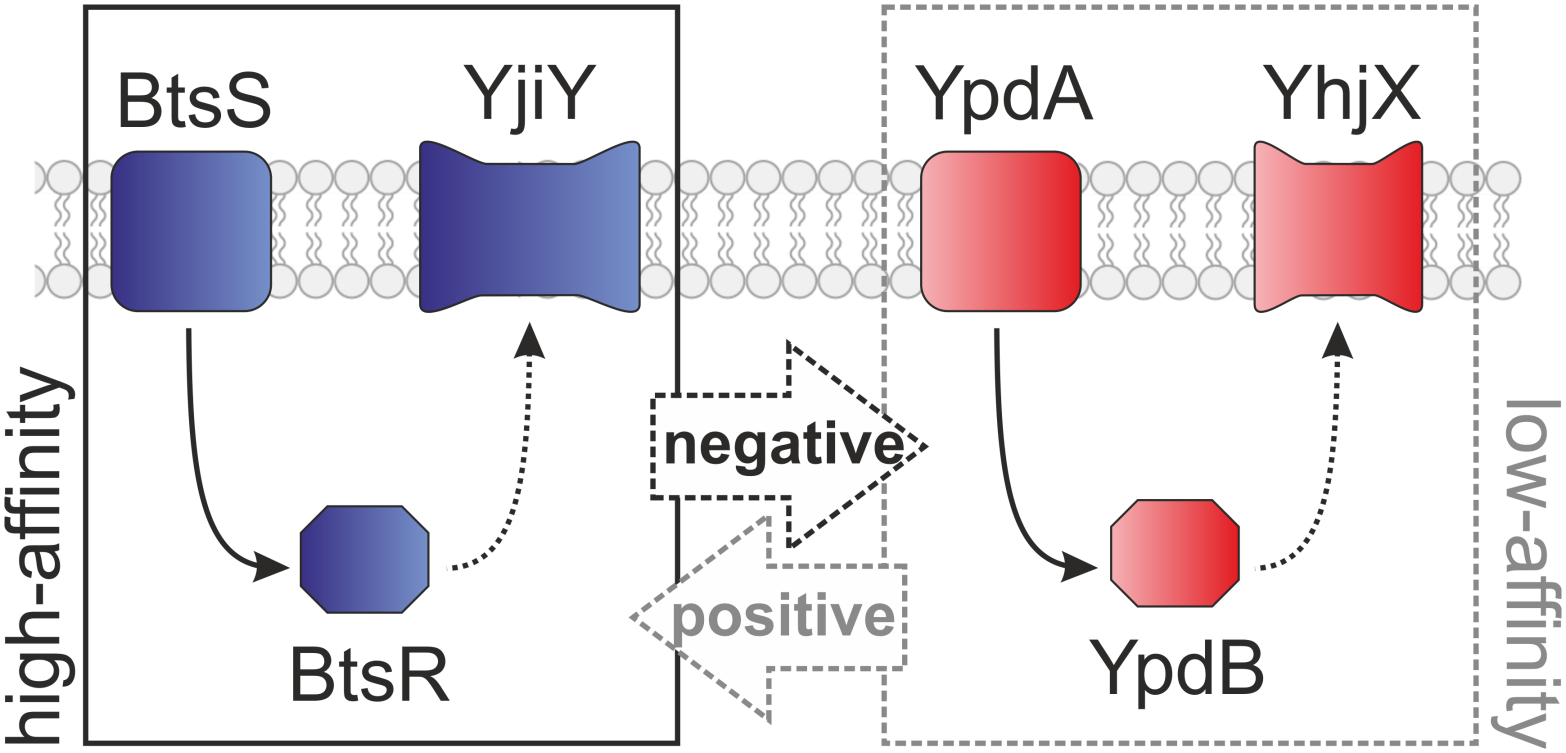
Schematic depiction of the functional links between the LytS/LytTR-type TCSs. The high-affinity BtsS/BtsR and low-affinity YpdA/YpdB TCSs are interconnected, which results in either the production of YjiY, a CstA-like transport protein, or YhjX, a putative antiporter of the MF superfamily. Dashed lines indicate predicted components and effects.

In *E*. *coli*, a signaling network including the TCSs BtsS/BtsR and YpdA/YpdB forms a pyruvate-responsive unit, which controls the expression of the two putative transporters YjiY and YhjX. Under nutrient limitation, the BtsS/BtsR system senses very low pyruvate concentrations (<5 μM) and subsequently activates *yjiY* induction [9]. The YpdA/YpdB system responds to external pyruvate with a lower affinity (>600 μM), which results in *yhjX* expression [7]. In *E*. *coli*, the functional link between the two systems provides for positive feedback from YpdA/YhjX to *yjiY* transcription, while *yhjX* is negatively regulated by BtsS/YjiY activity [8] (Fig 7). In *S*. *enterica* serovar Typhi (S. Typhi), the target gene of the BtsS/BtsR-type TCS has been identified as *cstA1* [17]; however, the YpdA/YpdB-type TCS is not encoded in this bacterium. We therefore investigated the distribution and co-occurrence of the two systems by comparative genomics, and discovered that the majority of γ-proteobacteria possesses only the BtsS/BtsR system, while YpdA/YpdB system is predominantly found in addition to the BtsS/BtsR system (Fig 2). Besides its central role in bacterial metabolism [18], pyruvate was recently linked to cell division [19], pathogenicity [20,21] and oxidative stress response [22,23], which emphasizes its functional versatility, and provides a rationale for the presence of more than one pyruvate-responsive signaling system in many bacterial species. The phylogenetic distribution of BtsS/BtsR and YpdA/YpdB provides the first indication that both systems might co-operate in a complementary manner. While BtsS/BtsR acts as a high-affinity pyruvate sensor under nutrient limitation [9] and is found in the majority of γ-proteobacteria, YpdA/YpdB functions as an additional, most probably low-affinity pyruvate-responsive system. The differential regulation of low- and high-affinity transporters for the same important substrate is a widely distributed phenomenon in all living organisms, and is associated with a competitive advantage [24]. The combination of a low- and high-affinity transport system was recently described for phosphate uptake in *Saccharomyces cerevisiae*, where individual cells express predominantly either a low- or a high-affinity transporter, each of which can confer a similar phosphate uptake capacity [25].

The DNA-binding motifs recognized by BtsR and YpdB in *E*. *coli* were found to be highly conserved, and were recognized in six out of eight (BtsR) and three out of five (YpdB), respectively, of the tested species (Figs 4 and 5). In contrast, the amino acid sequences of the signaling proteins were less conserved (Fig 3). There is an inverse correlation between the rate of evolution of transcription factors and the numbers of genes they regulate, and transcription factors that regulate many genes evolve more slowly than those with fewer targets [26]. This rule certainly holds for BtsR and YpdB of *E*. *coli*, because each controls one single target gene [7,10]. It is important to note that the domain organization of BtsS- and YpdA-type proteins was found to be conserved (Fig 3). Moreover, the sequence of the GAF domain is more highly conserved than others, and might therefore serve as a hub for an additional, as yet unknown intracellular stimulus. Indeed, GAF domains constitute one of the largest families of small-molecule-binding regulatory domains known [27].

Despite different degrees of conservation, our results suggest that the functionality of the BtsS/BtsR and YpdA/YpdB systems is conserved in most of the tested species of γ-proteobacteria. We were unable to identify the cognate BtsR and YpdB binding sites in *S*. *marcescens* and *A*. *hydrophila*, which would explain why we could not detect any promoter activity with the *E*. *coli*-based reporter system (see Figs 3–5). Nevertheless, we were able to detect external pyruvate at a minimal concentration about 220 μM in the medium (Table 2). In addition, C. *freundii*, *E*. *aerogenes*, *Y*. *enterocolitica*, and *V*. *harveyi* transiently release pyruvate into the medium, probably as result of their overflow metabolism. *X*. *szentirmaii*, *S*. *enterica*, *A*. *hydrophila* did not excrete pyruvate under the tested growth conditions, and *S*. *marcescenes* excreted pyruvate at a later time during growth. Although these results clearly indicate differences in the metabolic behavior of the various γ-proteobacterial species, these data support our hypothesis, according to which YdpA/YpdB responds to pyruvate with low affinity, while BtsS/BtsR responds with high affinity to pyruvate and consequently to nutrient limitation by a thus far unknown molecular mechanism.

The mammalian intestine is one of the most densely populated of all known microbial habitats. Species of the *Enterobacteriaceae* predominantly colonize the small intestine, which provides sufficiently high nutrient levels [28]. Pyruvate might be excreted as part of an overflow metabolism in order to prevent intracellular pyruvate toxicity. Under these exceptional conditions, low-affinity pyruvate sensing and subsequent metabolite uptake (mediated via YpdA/YpdB-type system) might function to replenish resources. On the other hand, the transition of the bacteria into the highly competitive colon confronts *E*. *coli* and other *Enterobacteriaceae* with conditions of nutrient limitation. This scenario requires the high-affinity pyruvate sensing BtsS/BtsR-type system and subsequent expression of a putative high-affinity transporter and furthermore generates selective pressure favoring its wider distribution.

## Supporting information

S1 Table List of all LytS-type HKs identified by a local alignment search based on the full-length sequence of *E*. *coli* BtsS. NCBI Accession Numbers display the NCBI identifier and are taken from the NCBI database. Proteins are grouped according to their level of sequence similarity to BtsS or YpdA and their species of origin.
(XLSX)
